# Symmetric Surface Acoustic Wave Tweezers Based on 128° YX-LN for Dynamic Manipulation of Particle Patterns

**DOI:** 10.3390/mi17060639

**Published:** 2026-05-22

**Authors:** Peng Zhang, Hongliang Wang

**Affiliations:** 1 Department of Electronic Engineering, Taiyuan Institute of Technology, Taiyuan 030008, China; zhangpeng1@tit.edu.cn; 2 State Key Laboratory of Extreme Environment Optoelectronic Dynamic Measurement Technology and Instrument, North University of China, Taiyuan 030051, China

**Keywords:** surface acoustic wave, acoustic tweezers, particle manipulation

## Abstract

In the fields of cell engineering, bio-fabrication, and targeted therapy, achieving high-precision manipulation of microparticles and cells remains a technical challenge. Although acoustic tweezers based on surface acoustic waves (SAWs) offer a promising solution, the structural complexity of conventional SAW devices has limited their practical applications. This work proposes a symmetric interdigitated transducer (IDT)-based acoustic tweezers device featuring a simple structure and high flexibility for modulating acoustic pressure field patterns and enabling particle manipulation. Theoretical investigations into the particle manipulation mechanism of the proposed device were conducted using the finite element method. A detachable polymethyl methacrylate (PMMA) assembly chamber was also designed. The effectiveness of the device was validated through dynamic and reconfigurable manipulation experiments using fluorescent polystyrene microspheres. Experimental results demonstrate that the proposed device can rapidly and precisely modulate SAW to achieve array-based manipulation of particle clusters, forming corresponding array patterns. Compared with conventional sorting methods, this device offers advantages including low cost, high precision, ease of operation, and good biocompatibility, making it suitable for large-scale manipulation of microparticles and biological cells. This technology has the potential to expand the application landscape of SAW and may emerge as a cutting-edge approach for directed cell assembly and culture.

## 1. Introduction

Particle manipulation technology is widely used in various fields such as directed tissue engineering [1], organic macromolecule assembly [2], and target delivery [3]. The high-precision and large-scale manipulation of particles to form patterns has become a key technology in diagnosis and treatment process in biomedical and clinical medicine [4]. At present, optical tweezers, electrical tweezers, magnetic tweezers, and acoustic tweezers can achieve ordered arrangement and distribution of biological cells and organic macromolecules. Optical tweezers and electric tweezers typically require high-power optical and electric fields, but high-intensity physical fields could generate heat and damage biomolecules and cells [5,6]. Magnetic tweezers require pre-magnetization treatment of the sample, which increases the risk of sample contamination [7]. Acoustic tweezers have been extensively studied due to their biocompatibility, low power, non-invasive, and non-contact manipulation methods [8,9]. Among them, acoustic tweezers based on SAWs have been widely employed for microscopic particle manipulation owing to their miniaturized size and concentrated energy characteristics, and have undergone rapid development in recent years.

Current SAW-based micromanipulation techniques are primarily limited to two-dimensional patterning and particle trajectory control. However, the restricted variety of acoustic pressure field distributions that can be generated by acoustic tweezers hinders their widespread adoption in practical applications. To address this limitation, extensive research efforts have been undertaken by both academia and industry, aimed at developing more precise methods for acoustic particle manipulation. Acoustic holography can generate specified acoustic pressure and phase field on the target plane, allowing for the reconstruction of pressure field and precise manipulation of particles in liquids and air [10]. But this technology cannot simultaneously manipulate a large number of particles for dynamic reconstruction [11]. The acoustic vortex technology can control the particle rotation speed inside the vortex beam [12], and also has a 3D acoustic trap for wavelength scale manipulation. However, it lacks dynamic particle suspension stability [13]. The IDT acoustic tweezers technology with independent control can achieve smaller volumes, more pattern arrangements, and greater manipulation capabilities due to its simple geometric shape and flexible directional combinations. Examples include spiral acoustic tweezers [14] and symmetrically arranged acoustic tweezers [15]. However, these devices feature complex structures that require independent control by multiple multi-channel signal generators [16]. Moreover, signal interference between adjacent interdigitated transducers (IDTs) can occur, leading to degradation of device performance. In order to solve the above problems, this work used traditional bulk LiNbO3 (LN) to design acoustic tweezers based on independent IDTs. The aim was to improve the uniformity of the acoustic pressure field of the tweezers device and enhance the performance of particle manipulation.

## 2. Theoretical Analysis and Scheme Design

### 2.1. Model Establishment

**Figure 1:**
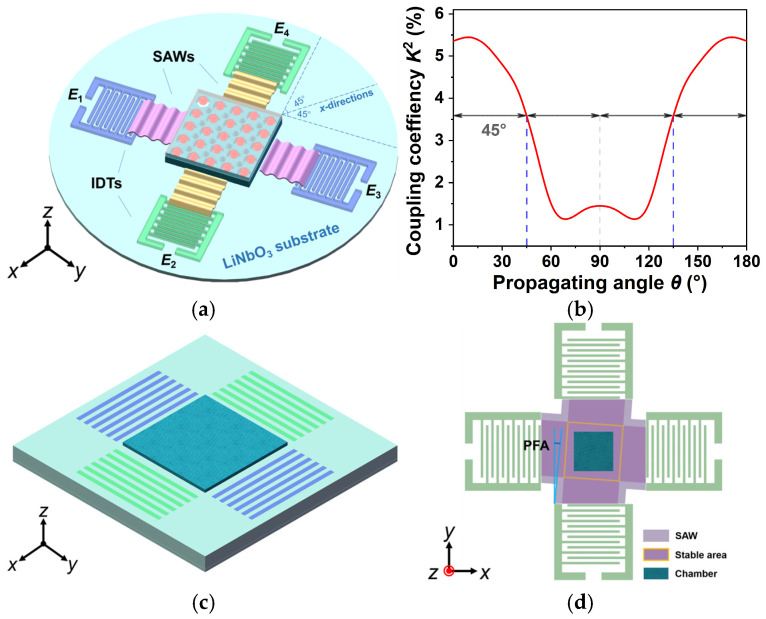
(**a**) Model diagram of symmetrical acoustic tweezers device. (**b**) Calculated *K*^2^ of the 128° YX-LN substrate. (**c**) 3D model used for the FEM simulation. (**d**) Design of the stable standing acoustic wave area used for the FEM simulation.

Dynamic acoustic pressure fields can generate time-variant acoustic radiation forces, thus providing the feasibility of cluster manipulation of particles in acoustic tweezers devices. In this work, 128° YX-LN was used as the piezoelectric substrate for the acoustic tweezers, and a symmetrical IDT acoustic tweezers device was designed on the LN wafer. The conceptual model is shown in Figure 1a. Its basic configuration consists of four uniform IDTs and a PMMA (polymethyl methacrylate) microfluidic chamber. Four IDTs, labeled *E*_1_, *E*_2_, *E*_3_, and *E*_4_, were arranged orthogonally on a piezoelectric substrate. The 128° YX-LN substrate is an anisotropic material, and the propagation characteristics of surface acoustic waves (SAWs) are influenced by the propagation angle. Figure 1b presents the variation curve of the electromechanical coupling coefficient (*K*^2^) as a function of the propagation angle. As shown in the figure, *K*^2^ values are identical at propagation angles of 45°, −45°, 135°, and −135°. To ensure that the acoustic wave intensities excited by the four IDTs are equivalent, thereby generating a periodically distributed acoustic pressure field within the chamber, the PMMA microfluidic chamber is positioned at the center region of the substrate. The angle between each IDT and the x axis is set to 45°, and all IDTs are placed at equal distances from the substrate center.

To investigate the working principle of the SAW-based acoustic tweezers device for particle manipulation, a finite element model was established using COMSOL Multiphysics 6.0, as illustrated in Figure 1c. The model parameters were adopted from [9], and the model consists of the LN substrate with Au electrodes deposited on it. A free tetrahedral mesh was employed in the finite element model, with a total of 2,937,489 degrees of freedom. The LN substrate had dimensions of 5200 μm × 5200 μm × 500 μm (thickness), and the water layer measured 2000 μm × 2000 μm × 10 μm (thickness). To ensure device operation, sufficient sedimentation of suspended particles/cells is required. Therefore, in the model, most particles/cells are positioned within the fixed pressure field at the bottom of the PMMA chamber. In simulation, the influence of the power flow angle (PFA) must be considered [17]. When SAWs propagate along the ±45° direction, there exists an approximately 4° angle between the group velocity and the phase velocity directions, corresponding to a PFA of 4°. The presence of the PFA caused the SAW coverage areas of the four IDTs to be incompletely overlapped. The region covered by only a single IDT was identified as an unstable region. Although the central region of the acoustic wave field mainly exhibited discontinuous phase variations, contact between the microfluidic chamber and the unstable region affected by the PFA was avoided [18]. Therefore, in this work, the central region characterized by a stable standing wave mode was specifically designed. Based on the PFA tilt angle, the effective excitation aperture, and the IDT spacing, the stable standing wave region for SAWs was determined, as indicated by the yellow rectangular box in Figure 1d. By ensuring that the dimensions of the microfluidic chamber are smaller than this stable region, interference of the PFA with particle manipulation can be eliminated, while simultaneously improving computational efficiency and simulation accuracy.

### 2.2. Principles and Analysis of Particle Manipulation

In an acoustic tweezers device consisting of uniform IDTs, when the wavelength *λ* is much smaller than the side length of the central chamber and the chamber height is relatively large, the acoustic pressure field inside the chamber typically exhibits an irregular three-dimensional distribution. The acoustic pressure field at the bottom center region of the chamber remains relatively stable and can be approximately regarded as a steady-state standing wave field. In contrast, regions near the chamber walls exhibit pronounced acoustic pressure gradients accompanied by significant traveling wave components, leading to the formation of acoustic vortices at the boundaries [19]. Since the present study focuses on the acoustic pressure field in the central region of the chamber, the peripheral acoustic scattering effects can be neglected.

**Figure 2:**
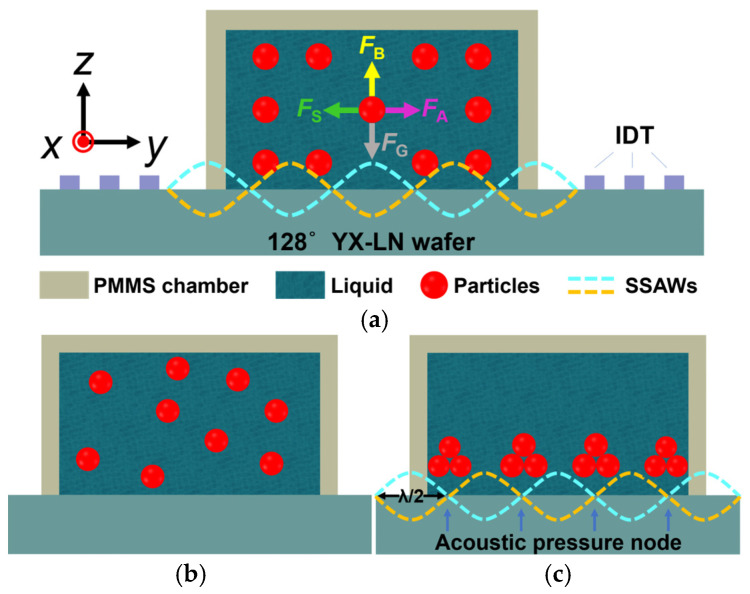
(**a**) Schematic diagram of force analysis in plane of particles inside the chamber. (**b**) Schematic diagram of irregular distribution of particles in IDT without working state. (**c**) Schematic diagram of particle pattern manipulation in acoustic field.

The acoustic radiation force (*F*_A_) was the core force enabling particle manipulation in acoustic tweezers devices. When acoustic waves propagated through a liquid, they created a spatially non-uniform acoustic pressure field. The difference in density and compressibility between the microparticles and the surrounding liquid caused the particles to scatter the acoustic waves. This scattering process altered the momentum distribution around the particles, thereby generating a net force on them, i.e., *F*_A_, which pushed the particles toward the nodes or antinodes of the acoustic pressure field and maintained them there. On the other hand, when acoustic streaming dominated in the flow field, the Stokes force (*F*_S_) emerged [20]. *F*_S_ was the viscous force resulting from fluid motion induced by the acoustic field and often led to non-specific aggregation or interference. When high-frequency acoustic waves propagated through a liquid, they lost energy due to viscous attenuation, and this lost momentum was converted into a steady direct-current fluid motion, giving rise to *F*_S_. When the liquid viscosity was high or the channel height was large, *F*_S_ tended to drag the particles, thereby disrupting the stable patterns formed by *F*_A_. Consequently, the relative direction and magnitude of *F*_A_ and *F*_S_ varied with the position within the chamber. *F*_G_ and *F*_B_ were static forces that determined the initial distribution of the particles in the absence of an acoustic field. Thus, the particles inside the chamber were primarily subjected to four types of forces: lateral *F*_A_ and *F*_S_, as well as vertical gravitational force (*F*_G_) and buoyant force (*F*_B_) [21]. A schematic illustration of the force model acting on the particles is shown in Figure 2a.

As shown in Figure 2b, in the initial state where no electrical signal is applied to the IDTs, particles are randomly dispersed in the liquid within the chamber. When an AC signal is applied to the IDTs, SAWs are excited on the LN substrate due to the piezoelectric effect. The IDTs oriented at four different angles generate SAWs propagating in multiple directions, which superimpose to form a dynamic standing surface acoustic wave (SSAW) field. The pressure fluctuations within this standing wave field exert *F*_A_ on the particles in the horizontal direction, driving them toward the acoustic pressure nodes. At these nodes, particle clusters are periodically assembled with a spacing of *λ*/2, achieving patterned manipulation of particles, as illustrated in Figure 2c. By configuring different coherent acoustic wave vectors, distinct acoustic pressure field distributions can be obtained, thereby enabling diverse particle manipulation patterns. Detailed discussions on this aspect are presented in subsequent sections.

## 3. Device Fabrication and Analysis

### 3.1. Device Fabrication and Assembly

**Figure 3:**
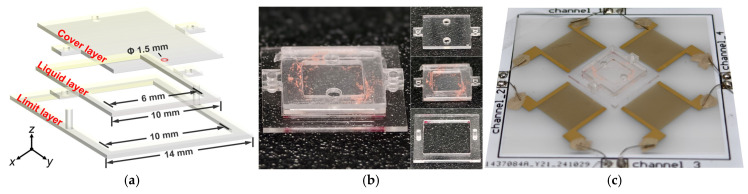
(**a**) Schematic diagram of detachable PMMA chamber structure model and parameter annotation. (**b**) Layer structure and assembly structure of PMMA chamber. (**c**) Local amplification of acoustic tweezers device.

The substrate of the acoustic tweezers device in this work is 128° YX-LN, and four uniform IDTs with symmetrical configurations were prepared on the substrate. The electrode material was Au and thickness was 200 nm. The IDT patterns were formed by using standard photolithography, and the electrodes were prepared using magnetron sputtering. The wavelength *λ* of IDTs was 400 μm, the aperture length was set to 25 *λ*, and the number of electrode pairs (*N*_i_) was 50. The central region of the substrate was a polymethyl methacrylate (PMMA) chamber. The chamber was designed as a detachable structure, divided into three layers: the cover layer with water injection holes, the liquid layer with limit holes, and the limit layer with limit columns. The model and key dimension annotations are shown in Figure 3a. The layer structure and assembly structure of the fabricated PMMA chamber are shown in Figure 3b. Considering the arrangement of IDTs and the length of the aperture, the geometric dimensions of the PMMA chamber were designed to be 6 mm × 6 mm to ensure that the chamber was completely covered by the SAW working area. To prevent adhesion of the cross-linked hydrogel, an anti-adhesion film was inserted between the substrate and the limit layer. Coupling oil was then applied under the film to facilitate acoustic wave transmission into the chamber and liquids. The fabricated symmetric acoustic tweezers device is shown in Figure 3c. Four IDTs were arranged orthogonally on the LiNbO_3_ substrate in a centrosymmetric manner, with the center-to-IDT line oriented at 45° to the x-axis. A detachable PMMA chamber was located in the central region.

### 3.2. Device Verification and Analysis

A sinusoidal signal with a frequency of 9.4 MHz and an amplitude of 6 V generated by a function signal generator was applied to the IDTs as the excitation signal to excite SAWs. The received signals were then detected and analyzed using an oscilloscope. For experimental convenience, the SAW device was connected to a PCB. To prevent the LN substrate from cracking due to heating, conductive silver paste was used for the interconnection. The assembly was then connected to the signal generator. For each pair of IDTs, one terminal was grounded, while the other terminal was connected to the input signal. The PMMA chamber was filled with deionized water, and the particle suspension was injected using a high-precision syringe at a volume ratio of 1:20 with respect to the deionized water. The suspension contained fluorescent polystyrene microspheres with a mass fraction of 2.5% and a diameter of 10 μm. The density of the PS particles was slightly higher than that of water, causing them to slowly sink in water. Moreover, the substantial difference in acoustic impedance between PS and water made the particles suitable targets for acoustic manipulation. Concurrently, a small amount of nonionic surfactant (Tween-20) was added to the PS particle suspension to mitigate particle aggregation. To enhance particle manipulation performance, the microparticles were allowed to sediment sufficiently before applying the excitation signals, ensuring that the majority of particles were distributed within the acoustic pressure field at the bottom of the chamber. The device was placed under a transmission microscope equipped with a camera for real-time observation of the particles. Finally, a micro-syringe was used to inject the PS microsphere suspension into the PMMA manipulation chamber. The focus of the microscope was adjusted until the microspheres in the suspension could be clearly and distinctly observed, at which point the adjustment was stopped. When the PS particle suspension reached a stable state, forming striped and dot-array patterns, images were acquired.

**Figure 4:**
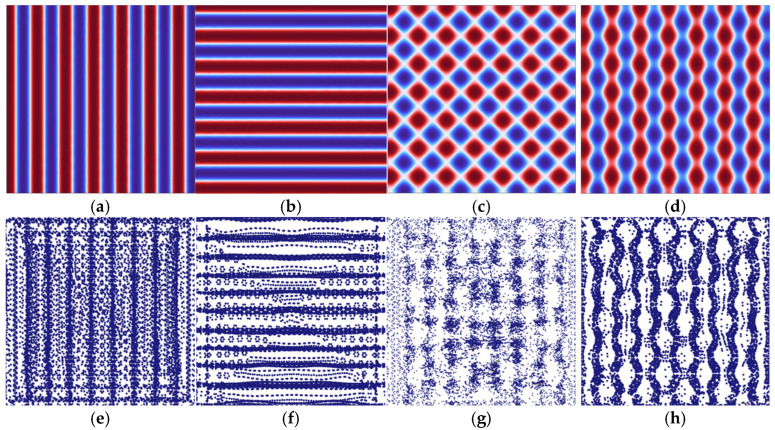
Simulated results of acoustic pressure fields when (**a**) *E*_1_ and *E*_3_ are actuated by the same excitation signal; (**b**) *E*_2_ and *E*_4_ are actuated by the same excitation signal; (**c**) *E*_1_, *E*_2_, *E*_3_ and *E*_4_ are actuated by the same excitation signal; (**d**) *E*_2_ and *E*_4_ input voltages are half those of *E*_1_ and *E*_3_. (**e**–**h**) Simulated results of particle trajectory prediction corresponding to (**a**–**d**).

The acoustic tweezers device designed in this work can generate and modulate SAWs along the alignment directions of the four IDTs. To achieve precise particle manipulation, the distribution of particles within the composite acoustic pressure field was analyzed based on the simulation model shown in Figure 1c. When a pair of IDTs with the same propagation direction (*E*_1_ and *E*_3_, or *E*_2_ and *E*_4_) are driven by electrical signals of identical amplitude and phase, a periodic acoustic pressure field oriented either horizontally or vertically is formed inside the chamber, as illustrated in Figure 4a,b. In the figures, the red and blue stripes represent the antinodes and nodes of the acoustic pressure field, respectively, with the distance between adjacent antinode and node being *λ*/2. When all IDTs are simultaneously excited with identical electrical signals, an acoustic pressure field featuring a periodic lattice structure is established within the chamber, as shown in Figure 4c.

Meanwhile, this work investigated the acoustic pressure field distributions generated by the two IDT groups under different electrical excitation signals. The amplitude of the SAW was controlled by adjusting the input voltage. Specifically, the input voltages applied to *E*_2_ and *E*_4_ were set to half of those applied to *E*_1_ and *E*_3_. Under this condition, a periodic striped array acoustic pressure field was formed inside the chamber, as shown in Figure 4d. Based on the analysis of the acoustic pressure field variation, a fluid-coupled particle motion model was further incorporated to predict the aggregation trajectories of the microparticles. The results are presented in Figure 4e–h, which show that the particle aggregation positions are generally consistent with the pressure nodes of the acoustic pressure field.

**Figure 5:**
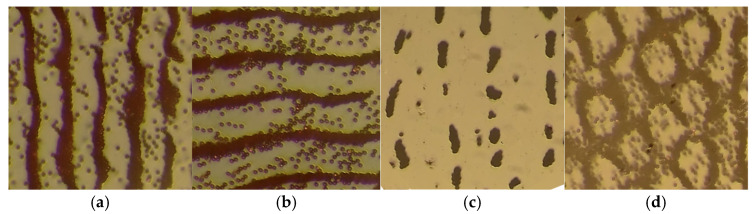
Experimental results of particle manipulation when (**a**) *E*_1_ and *E*_3_ are actuated by the same excitation signal; (**b**) *E*_2_ and *E*_4_ are actuated by the same excitation signal; (**c**) *E*_1_, *E*_2_, *E*_3_ and *E*_4_ are actuated by the same excitation signal; (**d**) *E*_1_ and *E*_3_ input voltages are half those of *E*_2_ and *E*_4_.

To validate the effectiveness of the designed model and the proposed particle modulation approach, experimental studies were conducted on the fabricated acoustic tweezers device following the procedures described above. Figure 5a–d present the rapid patterned manipulation of microparticles in vertical, horizontal, lattice, and stripe modes, respectively. The experimental results demonstrate that the majority of microparticles aggregate at the pressure nodes of the acoustic pressure field, assembling into the desired target arrays. It should be noted that the particle manipulation capability of the proposed acoustic tweezers device is not limited to the array patterns presented herein. By adjusting the AC signals applied to the IDTs, flexible design of arbitrary desired patterns can be achieved.

### 3.3. Discussion and Prospect

The effectiveness of the acoustic tweezers device was proved by simulation and experiment. The designed acoustic tweezers device used low-cost and easy-to-manufacture LN substrates, which enabled one-step photolithography, thereby greatly reducing costs, lowering fabrication difficulty and improving the success rate, and enabling mass production. The experimental results show that the proposed structure can manipulate the particle array by modulating the surface acoustic wave, and realize the corresponding array modes. Compared with the pioneering research [22], this paper added the technology of manipulating particles to form a complex array by modulating the input electrical signal, and designed a detachable test chamber. This is very important for imitating the natural tissue structure and the reconfigurable biological assembly of cells, and can be widely used in the culture of various specific cell arrays. Therefore, the technology is universal and can be used for large-scale operation of biomolecules and cells, which is expected to become a cutting-edge method for cell-oriented assembly and culture.

However, the limitations of this work need to be considered. Firstly, the stable region of the acoustic surface standing waves generated in the acoustic tweezers device is small, which limits the large-scale manipulation of biomolecules and cells. High-performance piezoelectric thin films can help to improve the piezoelectric properties of devices, which is the direction of future focus. In addition, due to the complexity and diversity of biological cells, including density, viscosity, cell activity, cell state, size and other factors, the accuracy, compatibility and biocompatibility of the designed acoustic tweezers device need to be improved. At the same time, the chamber environment is relatively simple, and the study of particle manipulation in chambers with complex structures will contribute to the further development of biological assembly technology. But in general, this technology has opened up a new field for the development of SAWs, which is helpful for the application of SAW technology in cell and molecular manipulation of organisms.

## 4. Conclusions

This work introduced the design and fabrication process of the acoustic tweezers device based on 128° YX-LN piezoelectric substrate. The devices realized various acoustic pressure field distributions in different shapes and arrangements, and can quickly and effectively manipulate particles/cells. The particle manipulation technology of acoustic tweezers devices is studied theoretically and experimentally by using the finite element method, and the PMMA assembly chamber with detachable structure is designed. Through the dynamic and reconfigurable operation of fluorescent polystyrene microspheres, the corresponding pattern array was realized. Furthermore, the technology of manipulating particles to form complex arrays by adjusting the input electrical signal is studied. The array patterns that can be formed include vertical array, horizontal array, dot array, adjustable stripe array, etc. This technology has the advantages of low-cost, high-precision, simple operation and great biocompatibility. It can be used for large-scale operation of particles and biological cells. It is suitable for frontier fields such as clinical medicine and tissue engineering. It is expected to become a frontier method for cell-oriented assembly and culture.

## Data Availability

Data will be made available on request. If you would like the research data for this paper, please do not hesitate to contact me at the address below.
